# Landscape ecological risk assessment of an ecological area in the Kubuqi desert based on Landsat remote sensing data

**DOI:** 10.1371/journal.pone.0294584

**Published:** 2023-11-16

**Authors:** Jie Zhang, Shulin Zheng, Yi Sun, Haijun Yue

**Affiliations:** Energy and Transportation Engineering College, Inner Mongolia Agricultural University, Hohhot, China; The University of Hong Kong, HONG KONG

## Abstract

Conducting ecological risk assessment of fragile ecological landscapes is a prerequisite for building an ecological security pattern and a necessary consideration for sustainable development. Engebei ecological demonstration zone is a typical ecologically fragile area located in the Kubuqi Desert. To explore the ecological status of Engebei, an ecological risk assessment model is used to assess its ecological risk, and the spatial correlation analysis is conducted based on the Moran index. The optimal grain size is obtained through grain size effect analysis, which is the foundation of landscape pattern analysis. The landscape ecological risk assessment model is constructed by the landscape indexes. Based on the division of small ecological risk zones, a spatial correlation analysis of ecological risks is conducted on Engebei. Results manifest that: (1) Overall, from 2005 to 2021, its spatial distribution features of landscape ecological risk level are relatively-high and high in the middle, and gradually reduce in the north-south direction, as shown below: the relatively-low ecological risk areas are widely spread, and the overall risk index decrease from 0.1944 to 0.1940; the area of low and high-level ecological risk areas show a decreasing trend, which decrease by 5.0102 km^2^ and 1.3132 km^2^ respectively; the area of relatively-low, middle, and relatively-high-level ecological risk areas increase by 0.2655 km^2^, 3.7803 km^2^, and 2.4852 km^2^, respectively. (2) The ecological risk value is correlated positively with spatial distribution, and the spatial aggregation forms are primarily low-low and high-high. (3) The ecological risk values in Engebei have a significant spatial correlation, and the spatial distribution shows a clustering effect, which is consistent with the spatial distribution. The study has certain reference value for the development and comprehensive regulation of ecological construction in Engebei, even in other ecologically fragile areas.

## Introduction

The combined impact of human activities and the natural environment directly affects changes in land cover. The alterations in regional land use have resulted in significant structural and functional changes in the original land ecosystem, influenced by external factors and internal succession [1]. Consequently, the ecological environment of the land has been greatly affected. Unreasonable land use methods and development intensity can cause damage to the regional ecological environment, particularly in ecologically fragile areas. Therefore, conducting research on the ecological risks associated with land use can help guide rational land planning, ensure the security of the ecological environment, and even promote sustainable regional development [2]. Regional ecological risk assessment is a field of study that focuses on analyzing the potentially dangerous conditions caused by both human and natural factors within a specific region [3]. This assessment utilizes interdisciplinary knowledge, including ecology, geography, and environmental science, to predict and evaluate the extent of these risks. Presently, ecological risk assessment approaches primarily revolve around analyzing risk sources, sinks, and landscape patterns [4, 5]. The ecological risk assessment, which is stemmed from risk sources and sinks, is commonly conducted using the approach of ‘risk-source recognition, receptor-analysis exposure, and danger evaluation’. By utilizing the ‘source-sink’ landscape and ecological risk assessment theory, the analysis of regional ecology’s risk reflects the availability and consistency of the ‘source-sink’ landscape theory [6]. The assessment of ecological risk based on landscape pattern directly describes and evaluates the regional ecological risk by focusing on the landscape pattern. This assessment method has gained significant attention in the field of landscape ecological risk assessment. For example, the ERI (Ecological Risk Index) based on LULC (Land Use and Land Cover) can intuitively and systematically assess the ecological risk of sub-Saharan Zanzibar [7]. The changes of some regional landscape elements can be evaluated by the theory of landscape ecology [8]. The ecological risk assessment approach can effectively depict the impact of land use change [9], and utilize landscape pattern indexes to indicate the changes in land utilization caused by urban expansion [10]. In terms of model construction, some researchers establish the ERA (Ecological Risk Assessment) model of susceptibility risk index relationship to evaluate the wetland ecological risk [11].

However, there have been limited studies conducted on the landscape ecological risk assessment in ecologically fragile zones with significant human disturbance, such as the Engebei mentioned in this study. The landscape pattern has the spatial grain characteristic of scale dependence, and the appropriate scale can improve the accuracy of landscape ecological risk assessment. On account of semi variogram [12], landscape pattern index [13], area information loss evaluation model [14], and other methods or models, the optimal analysis scale of the corresponding landscape can be effectively acquired.

The Kubuqi Desert, formerly referred to as the wilderness desert has undergone significant changes since 1989. A particular area within the desert, known as the Engebei ecological demonstration zone, exemplifies the ecological fragility of the region. Engebei was once a thriving place with plentiful water abundant vegetation, and stunning scenery. Unfortunately, due to worsening environment conditions and human activities, the land gradually transformed into a deserted area. Suffering from desertification and water and soil loss for a long time, Engebei had gradually become a sand sea. And its harsh natural conditions directly endangered the life and production safety of the Yellow River and the surrounding herdsmen. In 1989, Some volunteers went to Engebei for desertification control, opening the ecological construction there. Under the decades of governance, the ecological environment of the region has undergone apparent changes. Nowadays, it is also a demonstration base for biodiversity conservation and green development in China and a pilot area for China’s low-carbon land. Therefore, it is extremely important to obtain its ecological risk.

To this end, using the demonstration zone as an example, the research contents are: (a) Based on obtaining the appropriate analysis scale for landscape research, small ecological risk zones are divided for Engebei, becoming the foundation of the spatial correlation analysis of landscape ecology risk. (b) The landscape index is selected to establish the landscape ecological risk assessment model to carry out a related assessment [15] of the Engebei. (c) The spatial correlation analysis is conducted with GeoDa spatial analysis software, which analyzes the spatial aggregation model of ecological risks in this region. Grasping the primary status of ecological risk in Engebei can offer some reference foundation for scientific management and ecological improvement of the zone. In addition, it provides a research basis for further understanding the ecological environment status within the demonstration area.

## Material and methods

### Study area

**Fig 1:**
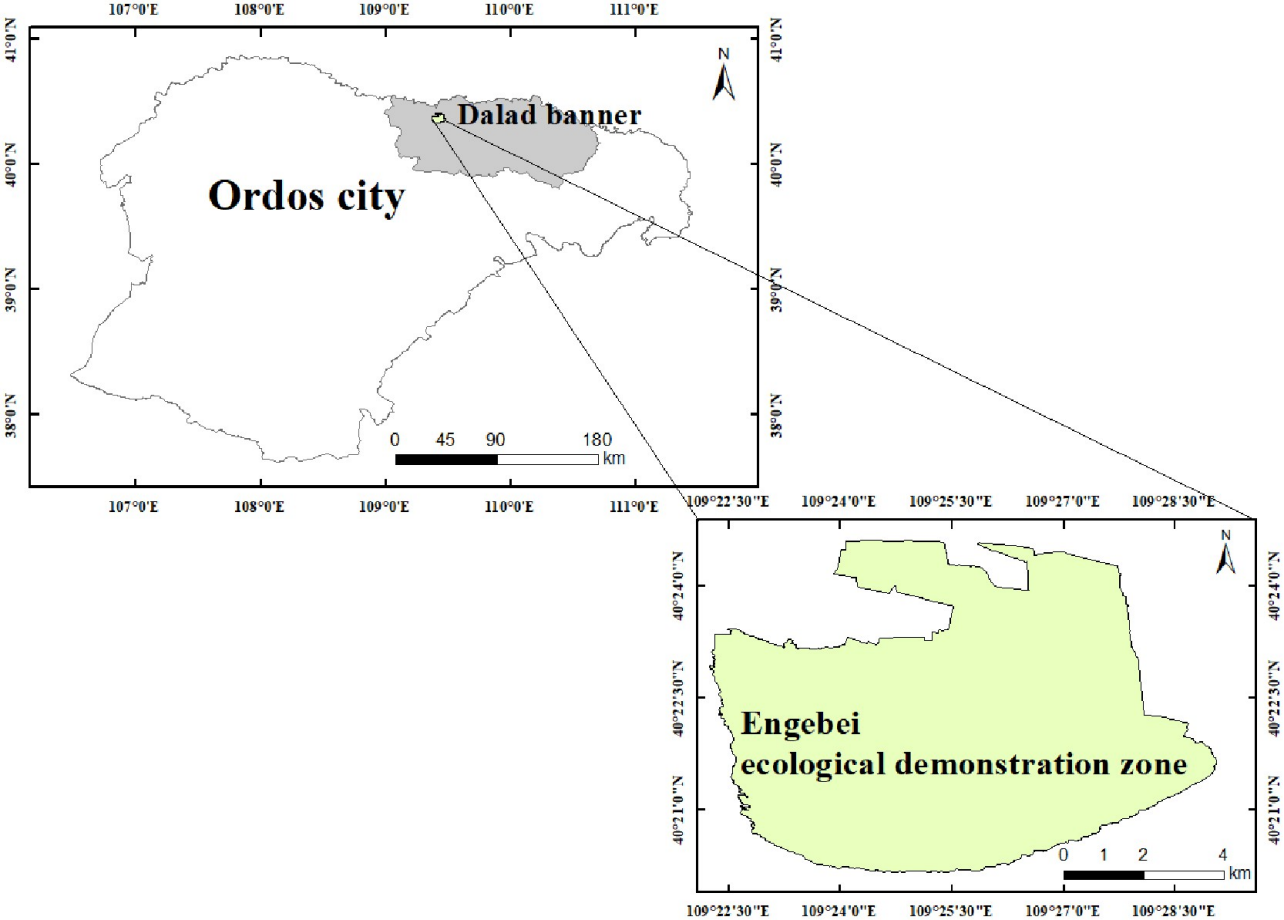
The study area: Engebei ecological demonstration zone. This figure is drawn using the shape files. The shape files are obtained from Resource and Environment Science and Data Center: https://www.resdc.cn/.

Engebei ecological demonstration zone (109°17′20′′–109°28′00′′E, 40°18′30′′–40°26′20′′N) (Fig 1) is located on Dalad banner, Ordos city, Inner Mongolia Autonomous Region, China, located in the south bank of the Yellow River and the middle section of the Kubuqi Desert, with an area of roughly 200 km^2^. The terrain of the whole region is low in the north and east, while high in the south and west. Engebei Valley runs through the demonstration area from southwest to northeast and flows into Heilai Valley. Its aeolian sandy is the elemental soil type there. Its plant type is dominated by sandy plants, some of which are woodlands and shrubs. The zone is adjacent to the Yellow River. It has a typical temperate continental arid climate, with an average annual temperature of about 7°C and an average annual precipitation of 250–300 mm.

### Data source and processing

The spatial resolution of commonly used land use products is relatively small. In addition, the study area in the study is small. Therefore, we use the Landsat satellite data with a higher spatial resolution to classify the land use types in this study. We choose data from the Geospatial Data Cloud (https://www.gscloud.cn/), including four phases of remote sensing image data which are Landsat5 TM data (July 5, 2005, July 10, 2010) and Landsat8 OLI_TIRS data (July 26, 2016, August 9, 2021), with a resolution of 30m. The average cloud cover is less than 5%, and the data quality is good. Since the time span of the study is 17 years, a spectral based image classification method, namely supervised classification, is chosen to classify land use types to ensure data availability. The supervised classification method used is SVM (Support Vector Machines). Meanwhile, considering the issue of different ground objects with the same spectrum in classification, transportation land is separated from construction land and classified separately. Some remote sensing data is selected that meets the following three requirements as the training samples for image remote sensing interpretation. The requirements are as follows: (1) the dates of the remote sensing data are as close as possible. (2) these training samples are representative. (3) the number of training samples meets the minimum requirements for establishing a classification discrimination function. The interpretation symbols are established by referencing Google Earth high-definition images from the same period, and combing with field survey results. The separability of the seven types of training samples obtained is greater than 1.9, indicating that the selected data has a good separability. The results indicate that supervised classification methods can be used. According to the GB/T 21010–2017 (2017 national land use classification system standard) and the above classification results, Engebei are divided into seven categories of land use which include grassland, cultivated land, construction land, transportation land, forest land, waters, and other land.

### Landscape index approach

The landscape pattern index method greatly concentrates the landscape pattern information, mirroring the landscape structure composition and part of the spatial configuration characteristics [16]. In the scale effect of the sensitive landscape indicators, the scale interval is the range where the index changes steadily. The area between the two inflection points is called the spatial grain domain. Six landscape indexes sensitive to particle size response and frequently used are selected to study the grain size effect of landscape pattern [17, 18]. They include PD (patch density), LSI (landscape shape index), AI (aggregation index), DIVISION (landscape separation), SHDI (Shannon’s Diversity Index), and SHEI (Shannon’s Evenness Index). The grain size effect is also called the granularity effect.

Landscape pattern has scale-dependent spatial grain characteristics. Some spatial information will be lost due to excessive large grain. Conversely, the overall law is easy to ignore because of too small grain. The conversion scale starts at 30m and ends at 500m. The grid scales are set at 20m and 50m intervals. 20m is the interval for the 30-270m section. 50m is the interval for the 270-470m section.

### Area information loss evaluation method

The area information loss evaluation can effectively evaluate the accuracy loss of vector data due to scale conversion, and evaluate the overall loss in a quantitative way [19]. And the formulas of the evaluation are as follows:

$${\text{L}}_{\text{i}}=\frac{{\text{A}}_{\text{i}}{\text{-A}}_{\text{bi}}}{{\text{A}}_{\text{bi}}}\times 100\%$$ (1)

$${\text{S}}_{\text{i}}=\sqrt{\frac{\text{1}}{\text{n}}}\times \sum_{\text{i}=1}^{\text{n}}{\text{L}}_{\text{i}}^{\text{2}}$$ (2)

where: L_i_ is the relative value of area loss; A_i_ is a certain landscape type’ area at a certain scale after data conversion; A_bi_ is the area of this type before scale conversion; n is the regional landscape types’ number; S_i_ is the whole region’ loss index.

We use 30m for land use classification, so the conversion scale starts from 30m. After simulation analysis, we find that when the grain size is 470m, there is a clear trend of regularity. Therefore, according to the area information loss evaluation method, we calculate the area loss index under 30-470m grain size in 2021, and then use the grain size as the abscissa and the land area loss index as the ordinate for mapping.

### Construction of landscape ecological risk assessment model

The landscape interference index describes the extent of external disturbance to the ecosystems under distinct landscapes, and the expression of the index is shown as follows [20]:

$${\text{E}}_{\text{i}}=\text{a}\times {\text{C}}_{\text{i}}+\text{b}\times {\text{F}}_{\text{i}}+\text{c}\times {\text{D}}_{\text{i}}$$ (3)

where: the E_i_ is the landscape interference index. The C_i_ is the fragmentation index, C_i_ = N_i_/A_i_. The F_i_ is the splitting index, ${\text{F}}_{\text{i}}=\sqrt{{\text{N}}_{\text{i}}/\text{A}}/\text{2}{\text{P}}_{\text{i}},\;{\text{P}}_{\text{i}}={\text{A}}_{\text{i}}/\text{A}$; The D_i_ is the dominance index, D_i_ = dL_i_ + eP_i_, L_i_ = N_i_/N. The letters a, b and c are the weight of C_i_, F_i_ and D_i_. N_i_ is landscape patches’ number. A_i_ is landscape type’s patch area. L_i_ is landscape type’s relative density. P_i_ is landscape types’ relative coverage. The weights of L_i_ and P_i_ are the d and e. N is landscape patches’ count. A is landscape’s gross area. In our previous studies, we find that the human disturbance activities about the study zone increased from 2005 to 2021, and the landscape fragmentation became greater. After 2016, the distance between patches became greater. The interference index’s distribution weights are a, b, and c, valuing 0.5, 0.3, and 0.2 [21, 22]. The distribution weights for dominance are the P_i_ and L_i_, valuing 0.6 and 0.4 [21, 22].

Landscape vulnerability is the fragility of ecosystems represented by diverse landscapes, reflecting the ability of anti-external interference. With the ability of landscape types to resist external interference being weak, their vulnerability and ecological risk is greater [23]. At present, the value of the index is attained by directly assigning values to different landscape types and normalizing. Using the expert scoring method, the vulnerability of landscape types in the zone is divided into seven ranks. Other land is the most vulnerable, so it is given the maximum value. And transportation land is the most stable, so it is assigned the minimum value. F_i_ (landscape vulnerability index) of each landscape type [24] (Table 1) is obtained after normalization.

**Table 1:** Landscape vulnerability index.

Landscape type	Assignment	F_i_
other land	7	1
waters	6	0.83
cultivated land	5	0.66
grassland	4	0.5
forest land	3	0.33
construction land	2	0.16
transportation land	1	0

The landscape loss index mirrors the extent of natural attributes of diverse landscape ecosystems when encountering natural and human disturbance [25]. Its formula is as the following [26]:

$${\text{R}}_{\text{i}}={\text{E}}_{\text{i}}\times {\text{F}}_{\text{i}}$$ (4)

where: E_i_ and F_i_ represent the interference and vulnerability index of the i-class landscape respectively.

The ecological risk assessment model of Engebei is constructed by the formula [27]:

$${\text{ERI}}_{\text{k}}=\sum_{\text{i}=1}^{\text{N}}\frac{{\text{A}}_{\text{ki}}}{{\text{A}}_{\text{k}}}{\text{R}}_{\text{i}}$$ (5)

where: ERI_k_ is the ecological risk index of the i-th-risk community; A_ki_ is the proportion of i-type landscape of the k-th-risk community; A_k_ is the k-th-risk community’s area; R_i_ is its i-category landscape loss index.

### Landscape ecological risk levels

The ordinary Kriging interpolation approach [28] and natural breakpoint method [29] are used to divide landscape ecological risks into five levels [30] (Table 2).

**Table 2:** Ecological risk classification.

Ecological risk level	Ecological risk value	Grade
Low-level	ERI<0.16	I
Relatively-Low-level	0.16≤ERI<0.23	II
Middle-level	0.23≤ERI<0.30	III
Relatively-high-level	0.30≤ERI<0.37	IV
High-level	0.37≤ERI	V

### Division of small ecological risk zones

The establishment of small ecological risk zones is a premise concerning ecological risk landscape assessment. The sampling area should meet 2–5 times the mean area of the landscape patches about the researching region [31, 32], which can sufficiently mirror synthetical landscape pattern about the sampling location. The risk zone division steps are as follows: (1) The landscape pattern grid map at the best scale of the study area is calculated to obtain the average value of the landscape patch area in the corresponding year. (2) According to the calculation results, the size of the risk unit is determined. (3) The grid is divided according to the equal interval sample extraction method, and the number of small risk zones in the study area is calculated.

The spatial correlation analysis of landscape ecology risk is conducted through Moran’s I (Moran index). LISA (Local Indicators of Spatial Association) clustering analysis is based on the similarity of data in geographic space to analyze the degree of data aggregation, using the local Moran’s I analysis. Through GeoDa software, ecological risk values about the research area are brought into relevant formulas to count global Moran’s I value in 2005,2010,2016, and 2021. And the ecological risk of the research zone is analyzed by LISA.

## Results

### Remote sensing data processing

**Fig 2:**
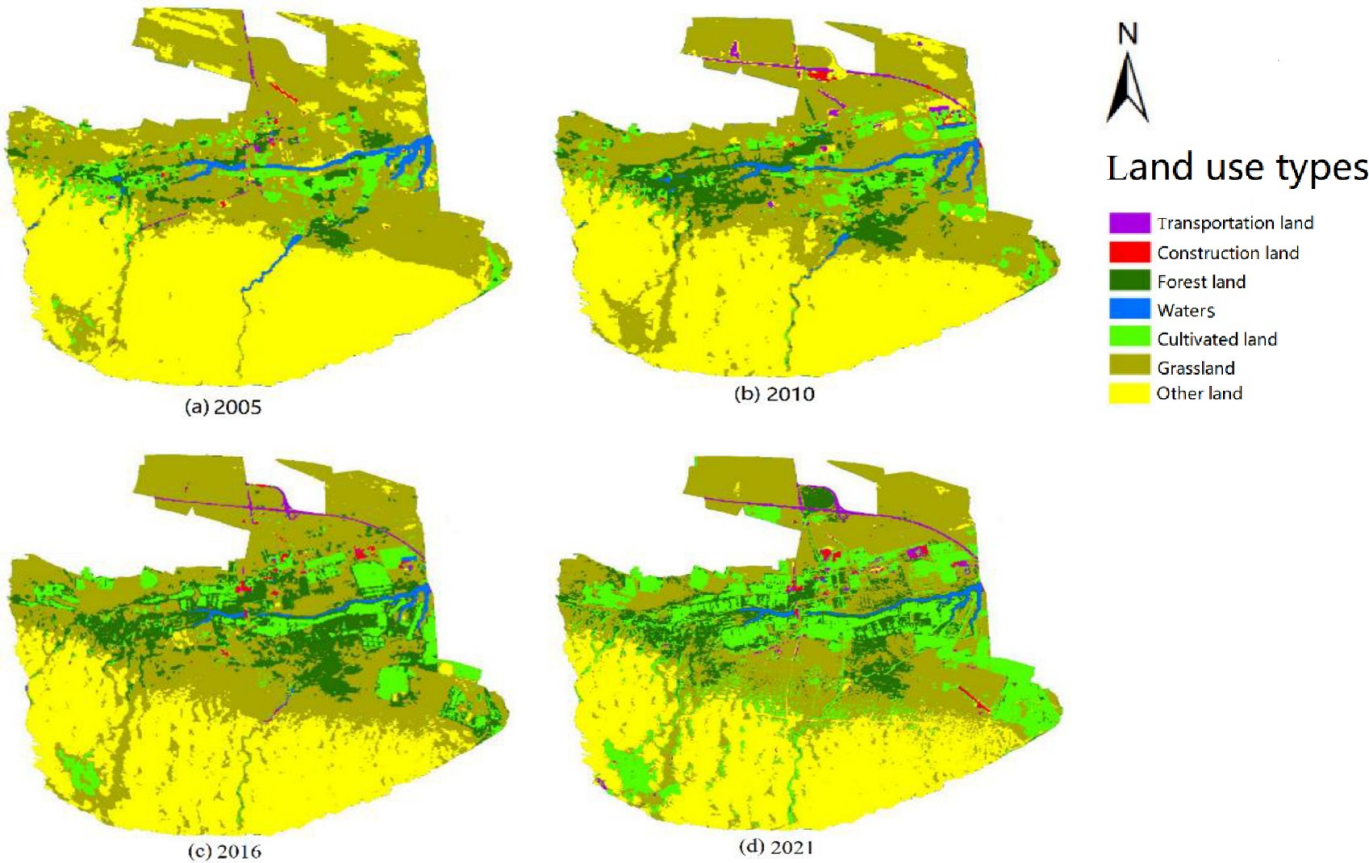
2005–2021 distribution of land use types in the study area. These maps are formed through supervised classification based on Landsat 8 satellite images and show the land use types of the region in 2005, 2010, 2016, and 2021. Imagery available from Geospatial Data Cloud: https://www.gscloud.cn/. (a)Land use types in Engebei in 2005. (b)Land use types in Engebei in 2010. (c)Land use types in Engebei in 2016. (d)Land use types in Engebei in 2021.

When interpreting the image of supervised classification results, land type which are with some errors in image interpretation and easily confused are adjusted. The data classification is completed through operations such as forestry sub class processing. Finally, the land use classification maps for each year are obtained (Fig 2). From 2005 to 2016, the area of forests and cultivated land shows an upward trend. From 2016 to 2021, some forest land has been converted into cultivated land. From 2005 to 2021, the waters show a downward trend, but the main waters in the central region remain relatively stable.

The selected validation samples are associated with the classification result map, and the confusion matrix method is used to evaluate the accuracy. Finally, the accuracy of the remote sensing supervised classification results is evaluated by the overall classification accuracy and Kappa coefficient (Table 3). The overall accuracy of the supervised classification results of the four remote sensing images is high, reaching over 85%. And the Kappa coefficient values are all above 0.8. Therefore, the classification effect of the supervised classification used meets the accuracy requirements of the research.

**Table 3:** Accuracy evaluation of landscape type interpretation.

Evaluation type	Classification image year			
	2005	2010	2016	2021
Overall accuracy (%)	86.96	91.52	96.71	93.36
Kappa coefficient	0.81	0.89	0.95	0.91

**Fig 3:**
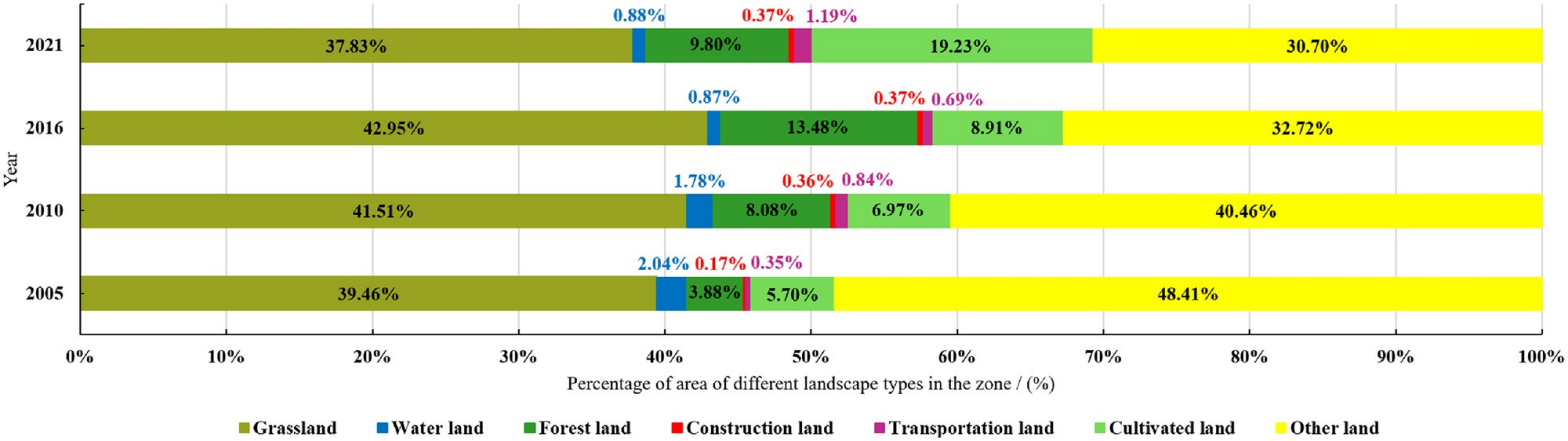
Percentage of different landscape types in the Engebei ecological demonstration zone from 2005 to 2021.

Based on classification, a landscape type structure change map of the study area is drawn (Fig 3). From 2005 to 2021, grassland and other land were the main landscape types in the study area. From the perspective of structural changes, the proportion of grassland and forest land first increases and then decreases; the proportion of arable land and construction land is gradually increasing. During the years, the construction land area remains stable after 2016; the area of transportation land first increases, then decreases, and then increases; the proportion of water bodies shows a downward trend from 2005 to 2016, and finally remains stable at around 0.88%.

### Spatial grain analysis of landscape pattern

**Fig 4:**
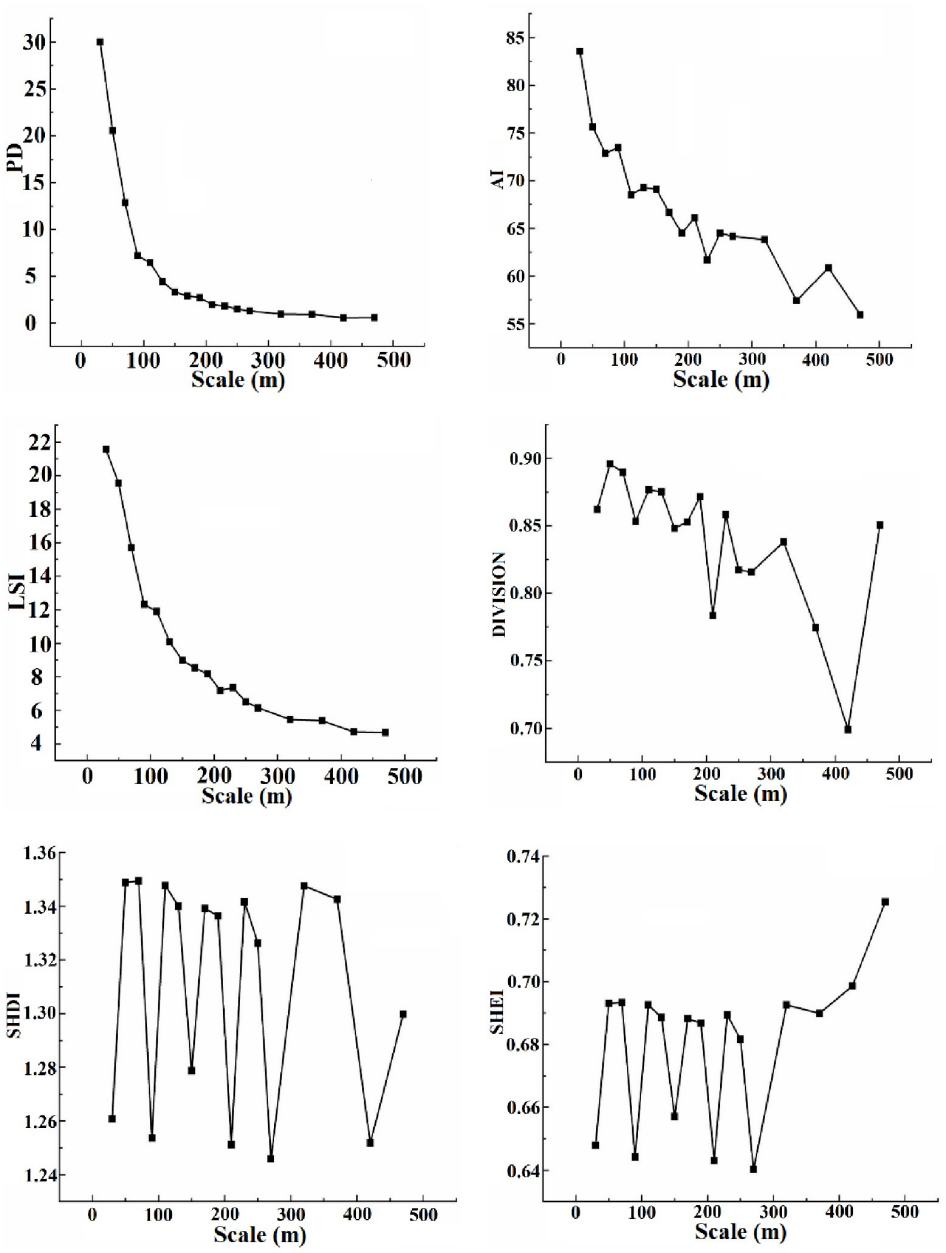
Grain size effect of sensitive landscape index. (a)PD (Patch Density). (b)AI (Aggregation Index). (c)LSI (Landscape Shape Index). (d)DIVISION (Landscape Spearation). (e)SHDI (Shannon’s Diversity Index). (f)SHEI (Shannon’s Evenness Index). According to Fig 4, the PD has inflection points at 90, 190, and 370m, and changes gently at 130~470m without obvious fluctuation. The LSI has obvious inflection points at 90 and 190m, and the change tends to be gentle at 190-470m. The AI has inflection points at 70, 110 and 150m, and has a significant downward trend when the particle size is greater than 50m. The DIVISION has inflection points at 50, 90, and 130m. SHDI and SHEI show similar sharp fluctuations at 30-370m, showing obvious grain size effect, and inflection points at 50, 90, and 130m. The closer SHEI approaches 1, the more uniform the types of landscape patches and the greater the diversity of the landscape. The value of SHEI is positively correlated with SHDI. When the scale changes from 50 to 100, the PD, AI, and LSI rapidly decrease, indicating that the degree of fragmentation of the landscape pattern decreases, the landscape becomes discrete, and the patch shape becomes simple. These indicate that the landscape distribution has become regular within this scale range. Therefore, when the scale ranges from 50 to 100, SHDI and SHEI change significantly. As the granularity effect curve tends to stabilize, the scale effect disappears. By synthesizing the particle size effect curves of these six landscape pattern indexes, there are turning points with significant changes, such as 50, 70, 90, 110, etc. Therefore, according to the scale effect analysis, the first-grain size domain is 50-70m, and the second-grain size domain is 90-110m. The research shows that the first-grain size domain is a better range for selecting the appropriate grain size [33], so the first-grain size domain 50-70m is selected as the selection range of the appropriate analysis grain size, that is, the appropriate analysis grain size domain.

This study is used the changes in six landscape pattern indexes in 2021 to determine the most suitable spatial grain interval. The vector data of 2021 obtained from the interpretation are rasterized. And then 17 grid maps with different scales are obtained. Finally, the grain-size-effect curves of landscape index in this study area are built, including PD, LSI, DIVISION, AI, SHDI, and SHEI (Fig 4). Consequently, the first-grain size domain and the second-grain size domain are determined by some inflection points in curves.

**Fig 5:**
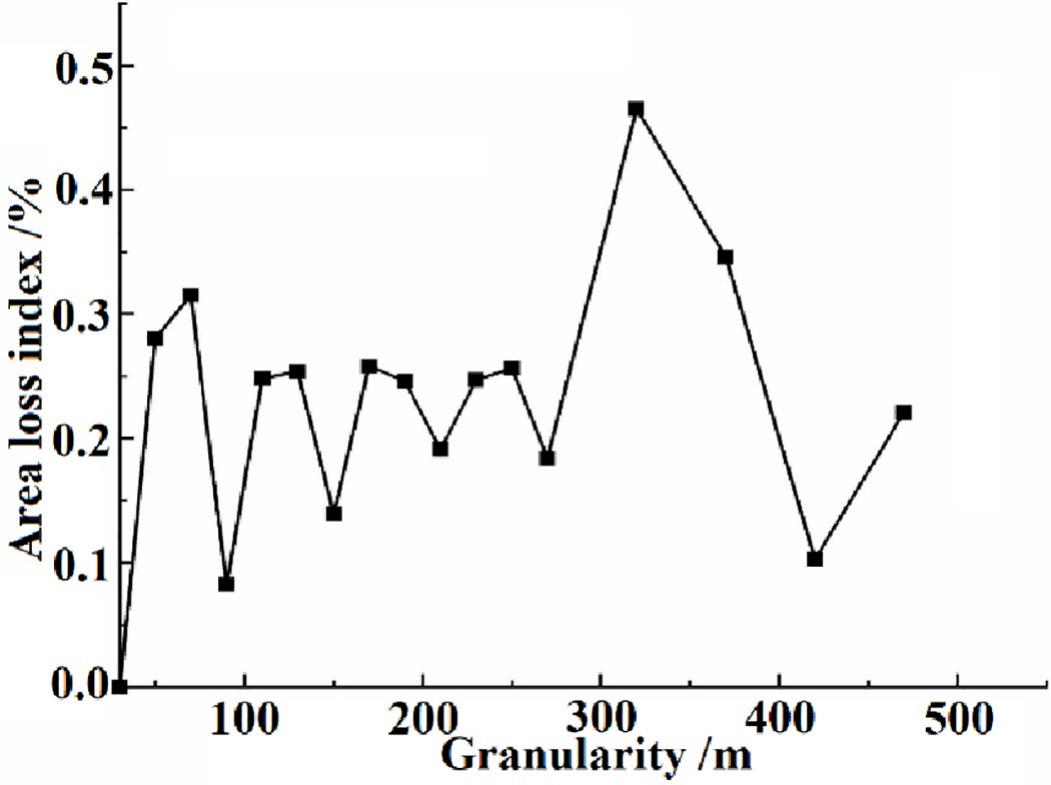
Regional land area loss index at different scales.

The area loss index at a particle size of 30 to 470m in 2021 is shown in Fig 5. At the conversion scale of 50m, 70m, 90m and 110m, the regional land area loss index is 0.28%, 0.31%, 0.08% and 0.24% respectively. On the premise that 50-70m is the appropriate analysis granularity domain, considering that the greater the regional land area loss index is, the worse the precision of the land area after the corresponding scale transformation is. Moreover, the landscape area loss index of 50 m grain size reaches the minimum value of 0.28%. Therefore, to obtain a higher precision transformation scale, 50m is selected as the appropriate analysis scale of the study area, that is, the optimal spatial grain.

### Trend of landscape pattern index change

**Fig 6:**
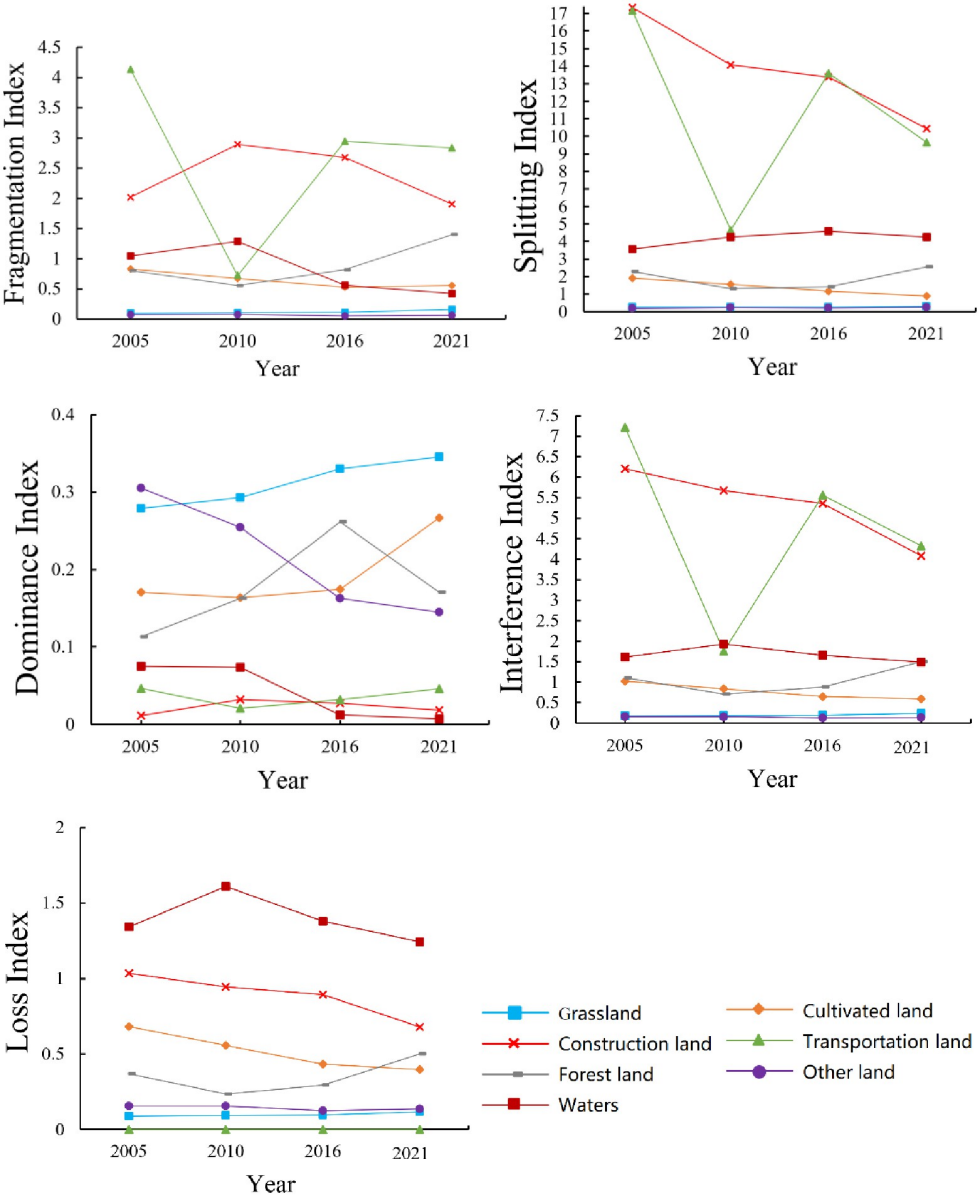
Landscape indices changes of diverse landscape types. (a)Fragmentation Index. (b) Splitting Index. (c) Dominance Index. (d) Interference Index. (e) Loss Index.

Different landscape indexes of seven land types changed from 2005 to 2021 (Fig 6). The fragmentation, interference, and loss index of the waters increase first and then decrease, and the decline of its dominance index drives the rise of its splitting index. The fragmentation, splitting, disturbance, and loss index of cultivated land decrease, while the dominance index increase. The fragmentation, separation and interference index of the transportation land show a fluctuating trend. The fragmentation, splitting, interference, and loss index of other lands maintain a stable trend, while their dominance index shows a downward trend. These indexes indicate that human interference has a significant impact on Engebei.

### Landscape ecological risk assessment

Based on the assessment model, the average values of overall landscape ecological risk concerning the research domain in 2005,2010,2016, and 2021 are 0.1944, 0.1920, 0.1704, and 0.1940. Over the years, the ecological risk value of the region has changed little. It shows that the ecological risk level of the region is relatively stable.

**Fig 7:**
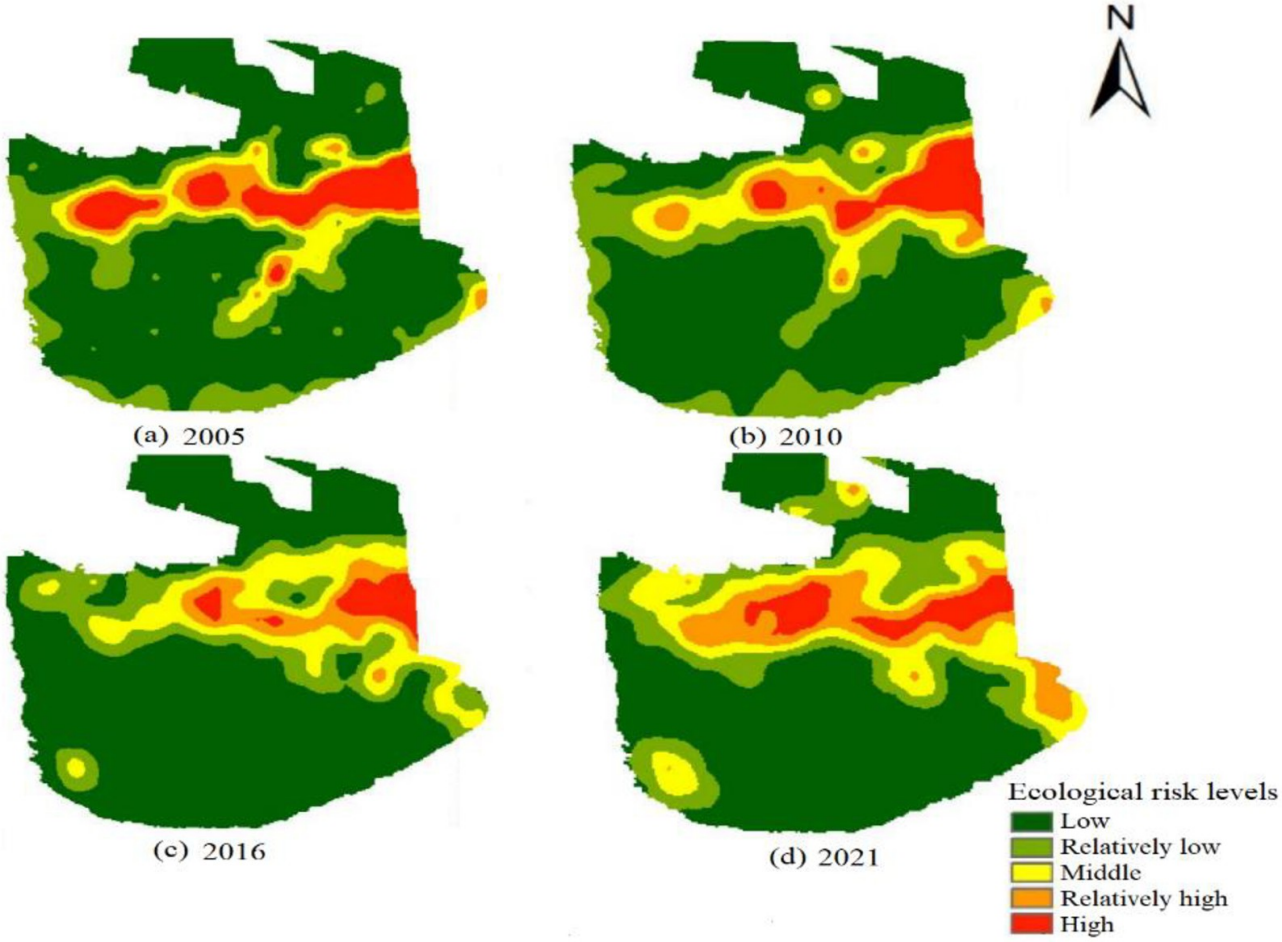
Distribution of ecological risk levels in the Engebei ecological demonstration zone. These maps are obtained by processing Landsat 8 satellite images. Imagery available from Geospatial Data Cloud: https://www.gscloud.cn/. (a)Distribution of ecological risk levels in Engebei in 2005. (b)Distribution of ecological risk levels in Engebei in 2010. (c)Distribution of ecological risk levels in Engebei in 2016. (d)Distribution of ecological risk levels in Engebei in 2021.

In terms of spatial distribution (Fig 7), high-level-risk areas are spread in Tianci lake and forest landscape intensive areas in the middle of the demonstration zone. The relatively-high-level-risk areas are spread around the high-level-risk, and its risk value is slightly lower than that of the high-level-risk areas. The middle-level-risk areas are primarily spread in the dense farmland. The low-level-risk areas are chiefly scattered in most of the southern region and the small north part of the demonstration zone. Overall, the middle part of this demonstration zone has a relatively high-risk level, gradually decreasing to its north and south. As a result, relatively-low-risk level areas widely spread in its north and south.

The areas of landscape ecological risk areas at all levels are counted (Table 4). From 2005 to 2021, the area of relatively-low-level ecological risk areas sightly increase, due to the proportions of relatively-low, middle, and relatively-high levels ecological risk proportions expand. The proportion of middle-level ecological risk areas scales up year after year. The major reason is that people have carried out development and utilization for a long time in the demonstration area, which has intensified the fragmentation of farmland and forest landscape. The proportion of relatively-high-level ecological risk areas rises totally, but its proportion cuts down to some extent in 2016 due to some areas transfer to middle-level ecological risk areas. And by 2021, its area was 5.0016 km^2^, accounting for 9.55%. The proportion of high-level ecological risk areas falls first and then goes up. From 2005 to 2016, it is in a downward stage, and the area proportion decreases from 7.69% to 3.04%, which decreases by 2.4273 km^2^. From 2016 to 2021, the cultivated land landscape in the central research area grows because of the interference of human activities. As a result, its land fragmentation and vulnerability become slightly greater. And the extent of high-level ecological risk areas scales up from 3.04% to 5.16%, an increase of 1.1141 km^2^. From 2016 to 2021, the area of relatively-low-level ecological risk areas expands, because the area of cultivated land in the central region increases. During this time, the land of Engebei is flat, and its landscape fragmentation is low, so the anti-external interference capability about the zone improves.

**Table 4:** Ecological risk grade area and proportion in Engebei.

Ecological risk level	2005		2010		2016		2021	
	Area /km^2^	Proportion /%	Area /km^2^	Proportion /%	Area /km^2^	Proportion /%	Area /km^2^	Proportion /%
Low	33.039	63.32	31.0563	59.52	34.0173	65.22	28.0288	53.51
Relatively low	8.7921	16.85	10.9512	20.99	7.7481	14.85	9.0576	17.29
Middle	3.8133	7.31	4.4568	8.54	6.2127	11.91	7.5936	14.50
Relatively high	2.5164	4.82	2.763	5.30	2.5956	4.98	5.0016	9.55
High	4.014	7.69	2.9475	5.65	1.5867	3.04	2.7008	5.16

### Spatial correlation of landscape ecology risk

**Fig 8:**
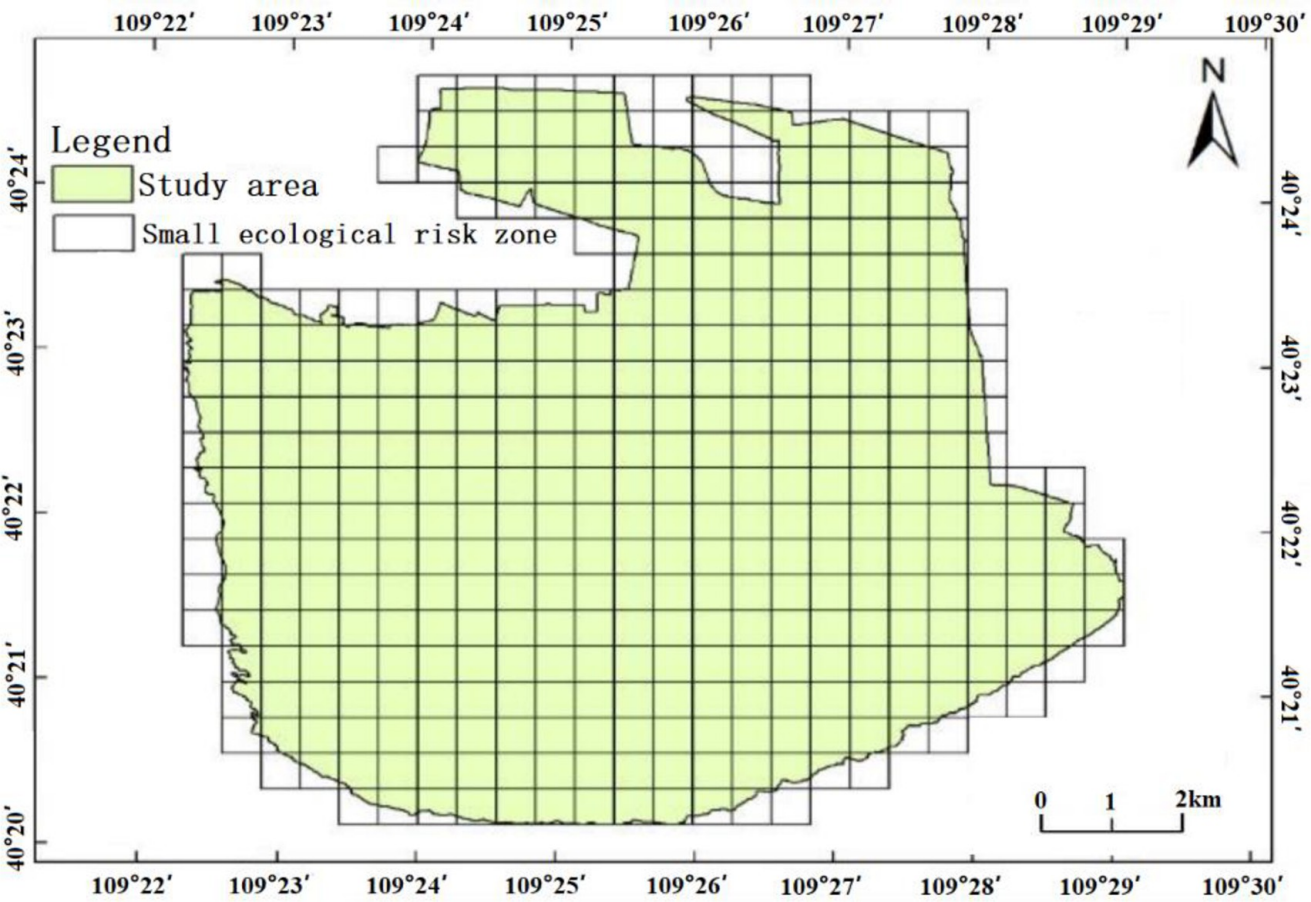
Engebei Ecological demonstration zone ecological district division. This figure is drawn using a shape file. The shape file is obtained from Resource and Environment Science and Data Center: https://www.resdc.cn/.

By calculating the landscape pattern grid map at the best scale about the researching area, the average size of landscape patch area in 2005, 2010, 2016, and 2021 is 0.052km^2^, 0.048km^2^, 0.043km^2^, and 0.033km^2^ respectively. The selected range of side length of risk cell is 0.322–0.408km. The division of cells should ensure enough number of units reflecting the landscape pattern distribution in the zone, and avoid problems such as calculation intensity and accuracy. Therefore, the 0.4km*0.4km risk unit is used for the estimated cell. The grids are divided with the equal interval method of sample extraction. Finally, the demonstration area is divided into 504 risk areas (Fig 8). And taking the grid as the center, we take samples to build the landscape ecological risk indexes of the demonstration zone.

**Fig 9:**
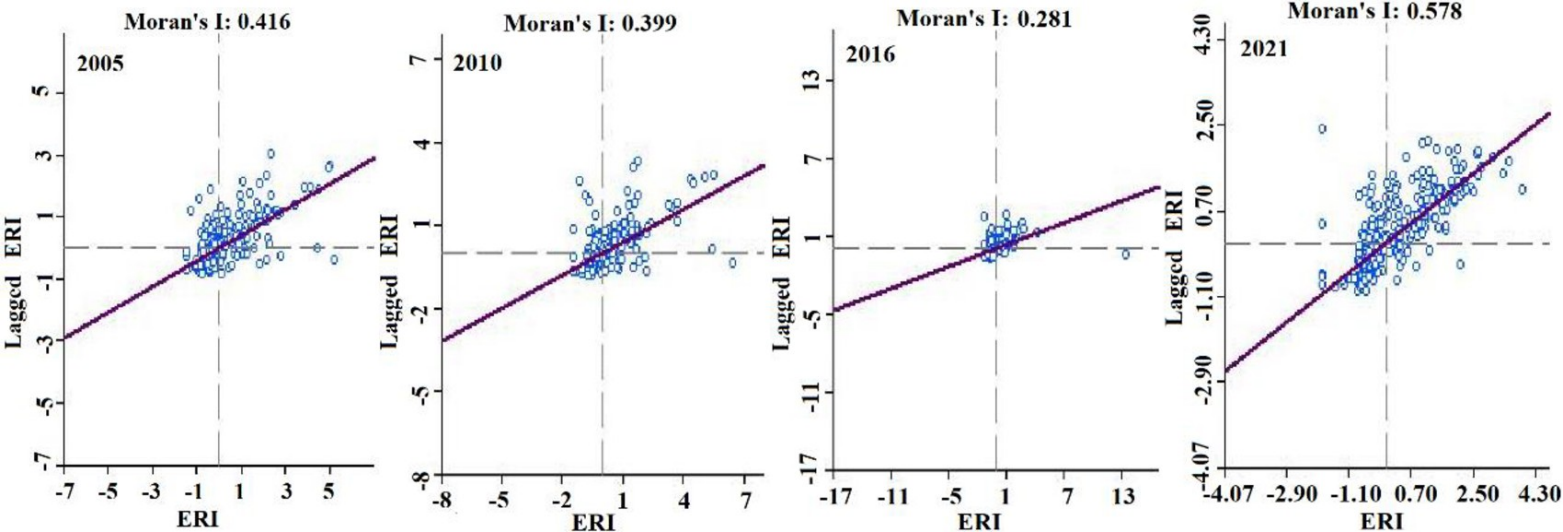
Scatter plot of Moran ’I index of ecological risk in Engebei. (a)The Moran Index of ERI (Ecological Risk Index) in 2005. (b) The Moran Index of ERI in 2010. (c) The Moran Index of ERI in 2016. (d) The Moran Index of ERI in 2021.

The values of global Moran’s I in 2005,2010,2016, and 2021 are evaluated 0.416, 0.399, 0.281, and 0.578 (Fig 9). The calculation results are all positive. They reflect the positive correlation among the spatial spread of landscape ecological risk values about the research region, indicating that the risk values present the characteristics of agglomeration in space [34]. And the aggregation of ERI decreases firstly and then increases in general.

**Fig 10:**
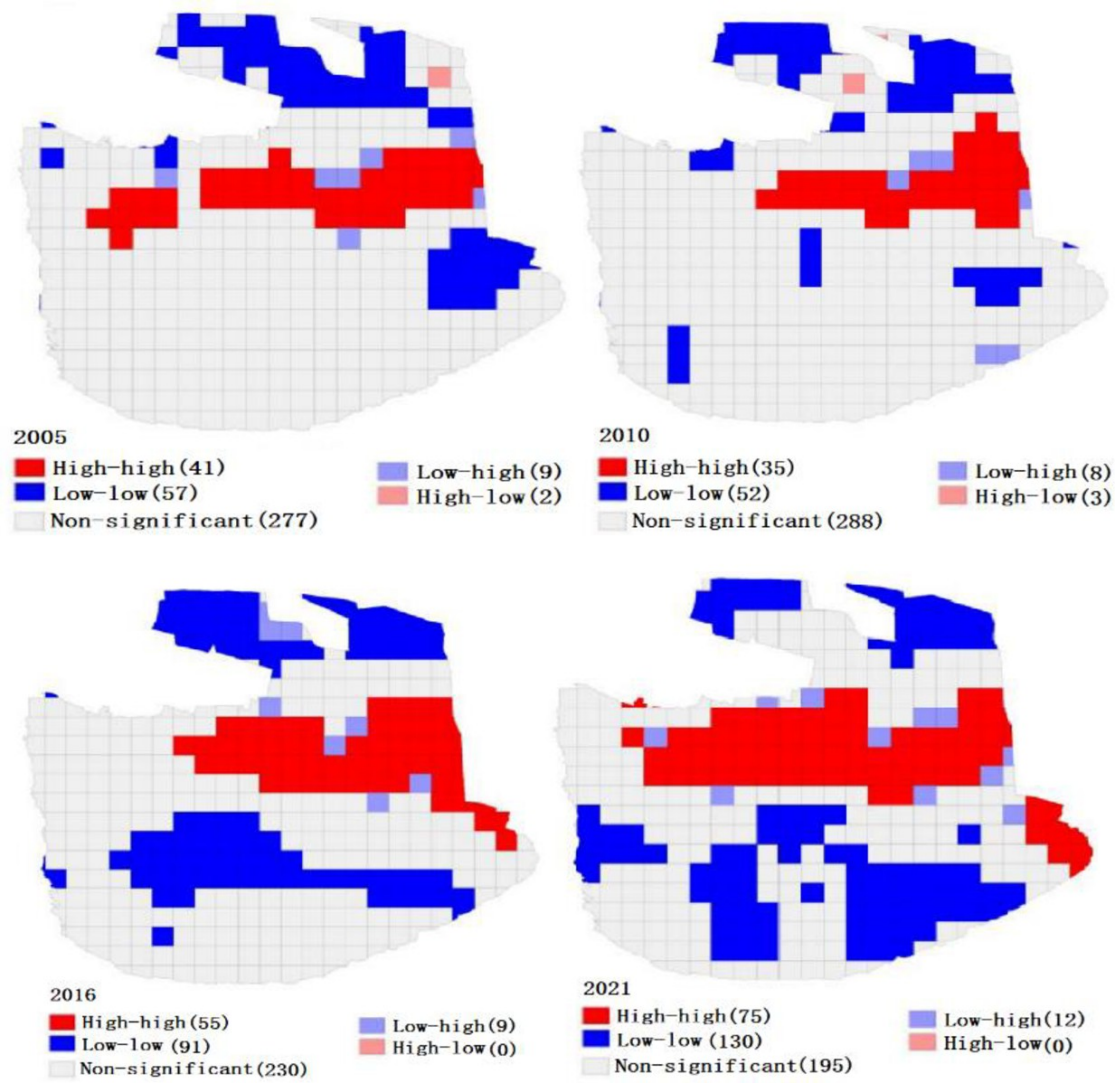
Local spatial autocorrelation of ecological risk in the Engebei. This figure is drawn using a shape file. The shape file is obtained from Resource and Environment Science and Data Center: https://www.resdc.cn/. (a)The spatial morphological aggregation of Engebei in 2005. (b)The spatial morphological aggregation of Engebei in 2010. (c)The spatial morphological aggregation of Engebei in 2016. (d)The spatial morphological aggregation of Engebei in 2021.

During the study period, the spatial morphological aggregation in Engebei is mainly characterized by non-significant aggregation, low-low aggregation, and high-high aggregation (Fig 10). As shown in the LISA analysis result graph (Fig 10), the spatial aggregation patterns of ecological risk in the area are primarily low-low and high-high aggregations. And its proportions of high-low and low-high aggregations are small. Low-low aggregation areas are mainly distributed on both sides of the south and north. High-high aggregation areas are primarily distributed in the central region. The number of low-high aggregation types exhibits an upward trend. From 2005 to 2021, the number of high-low autocorrelation types decreases, and the number of non-significant-spatial-autocorrelation-decreased by 82. By observing Figs 7 and 10, the high-high clustering areas are mainly high-risk-level and relatively-high-risk-level areas. The low-low concentration areas are mostly located in areas with the low-risk level. The insignificant clustering areas are primarily low-risk-level areas, with a small portion being areas with relatively-low-risk levels and middle-risk levels.

## Discussion

### Analysis of remote sensing data usage

The great growth of remote sensing technology makes remote sensing images widely exploited in landscape ecological risk analysis. Among various remote sensing images, Landsat image is extensively used because of its high spatial resolution, spectral resolution, positioning accuracy, and rich information. For example, through remote sensing interpretation of Landsat image, landscape ecological risk index can be established to dive into the temporal and spatial changes of landscape ecology risk [35, 36]. Thereby, this study is used such remote sensing images to study the landscape ecology risk in the ecologically fragile area, that is Engebei ecological demonstration zone. Zhang et al. [37] used SVM to classify land use types based on Landsat remote sensing data obtained during their research on landscape pattern changes, with an overall classification accuracy of over 85%. By SVM of remote sensing images, the land use type in this study area can be effectively classified into seven types (Fig 2), and the overall accuracy of the classification results is above 86.9% (Table 3). An overall accuracy of over 85% indicates that the classification results in this study are reliable. Based on classification, the land use structure change maps (Fig 3) drawn are also helpful for subsequent landscape pattern risk assessment analysis. Landsat satellite data is suitable for research on land use. Darren et al. [38] used Landsat data to evaluate the impact of land use/land cover changes on surface temperature in Cameron Highlands. Liu et al. [39] obtained land use data using Landsat data by supervised classification, conducting research on the spatio-temporal patterns, and driving forces of land use under urbanization in China.

### Analysis of the grain size effect

The grain size effect is the basic content of landscape pattern research. Scale maps can be used to analyze the landscape scale effect of different vegetation types at various space-time scales. Zhou et al. [40] quantify the granularity effect of landscape indexes using the landscape pattern index and variation coefficient, constructing an information entropy model to determine the optimal granularity. In this study, we choose two methods to obtain the optimal spatial grain of the study area, including landscape index approach and area information loss evaluation method. And these two methods are relatively simple to implement. Some researchers calculated landscape pattern indexes for various land use types through one year of remote sensing data, obtaining the spatial scales required for their research [18, 41, 42]. When selecting the optimal granularity, Hu et al. [18] select landscape pattern indexes such as SHEI, SHDI, AI, and DIVISION, and combine the landscape index approach and area information loss evaluation method. Similarly, we calculate some landscape pattern indexes from 2021 remote sensing data to obtain the spatial scale needed for landscape ecology risk assessment. And we determine the best spatial grain with the help of area loss index. The difference is that we have added two additional indexes, PD and LSI. Among them, the PD can reflect the degree of fragmentation of various land features, and the LSI can reflect the complexity of landscape shape changes. According to the Fig 4, each landscape type in Engebei has a significant granularity effect on the selected landscape index. Under the premise of the first scale domain, 50m is selected as the optimal spatial grain of the study area.

### Analysis of landscape pattern index

Since the implementation of ecological construction in the 1980s, generations have traveled to Engebei for sand prevention and control, resulting in a vegetation coverage rate of 78% and a forest coverage rate of 41% [43]. More than 7 million trees, 50000 acres of shrubs, and 5000 acres of excellent grass are planted in the demonstration area. The number of animal species has also increased from over 20 to over 600. The biological chain and population of animals and plants have been effectively restored. The improvement of its ecological environment has also driven the development of the sand industry and tourism industry, and has become a national demonstration city for ecological tourism construction.

Zhou and Luo [44] construct a comprehensive ERI using the landscape interference index, vulnerability index, and loss index. Similarly, we utilized these indexes to construct an ERI for ERA model. The demonstration area is a transitional zone of desert and arid grassland. After artificial afforestation, fragmentation and interference index of grassland and forest land show an upward trend. Because grassland is easier to survive than forest land, the dominance index of grassland shows an upward trend, while the dominance index of forest land is on the rise first and then on the decline. Due to the inherent geographical advantages, the splitting index and loss index of grassland show a stable trend. Because the survival rate of forest land is not high, its loss index decreases first and then increases. Due to the greater vulnerability of the waters, human interference must be appropriate.

Through the landscape index approach, we find:

(1) As the construction and development of the demonstration zone push forward people’s living improving, travel and entertainment needs, the splitting, interference, and loss index of construction land are getting hitched. (2) As the demonstration area seated in the transitional belt of desert and arid grassland, the dominance index of grassland shows an upward trend. (3) Because the survival rate of artificial afforestation under the aeolian sandy soil environment is not high, the dominance index of forest land increases first and then decreases. (4) As Tianci lake (also called the Engebei ditch, a large ditch with a length of 2500 meters, a width of 100 meters, and a depth of over 20 meters, see Fig 2) and other waters drove the ecological development of the demonstration area, the water splitting index increases. The dominance of cultivated land is also on the rise. (5) The dominance index of various land types is on the upswing, manifesting that the spread of land types of the demonstration zone is relatively apparent.

### Analysis of landscape ecological risk assessment

Introducing the landscape ERI to study the evolution of landscape ecological risks from the perspective of landscape ecology can provide a reference for the construction of ecology security patterns in the research area [45]. Tan et al. [46] constructed regional landscape ERI to evaluate the landscape ecological risk driven by land use transformation. This study utilized landscape ERI to evaluate the ecological risks of Engebei’s landscape under human interference.

Combining Figs 2 and 7, we find that the low-risk-level areas are mainly grassland and other land, while the middle-risk-level areas are mostly farmland and forest land. The relatively-high-risk-level areas and high-risk-level areas are located at the central part of the demonstration area, where forests, grasslands, and water bodies are distributed.

Through the research on remote sensing images of landscape ecology risk assessment in 2005, 2010, 2016, and 2021, the governance of the demonstration area still needs to combine the long-term and short-term planning of ecological management [47]. The following construction suggestions have been proposed for different levels of ecology risk zones in the research area:

(1) The high vulnerability of the water landscape and the fragmentation of forest landscape patches make the middle of the zone the highest landscape ecological risk. Attention should be paid to ecological restoration here, such as planting plants suitable for growing in deserts. Desert plants generally have specialized structures and functions that adapt to adverse conditions such as drought, high cold, and barrenness [48]. (2) Compared to the area of high-risk-level and relatively-high-risk-level areas, the area of middle-risk-level areas is relatively large. And the middle-risk-level areas are mainly distributed near or around these two types of risk areas. It is necessary to strengthen the dynamic monitoring of ecological security in middle-risk-level areas, prevent these areas from transforming into high-risk-level areas, and carry out targeted ecological engineering. Presently, the land use in this type of area is relatively single. For example, the cultivated land is spread mostly in blocks in middle-risk-level areas. (3) The low-risk-level areas are chiefly scattered in the sandy landscape, with continuous and relatively complete landscape patches. As shown in Fig 7, with human interference, the area of low-risk-level areas tends to decrease. With the disturbance of human activities being small, there is a relatively low-level ecological risk. Therefore, it is necessary to combine previous governance plans and continue to choose appropriate ecological governance technologies to promote the ecological development of the research area. And it should ensure the effective combination of natural restoration and artificial governance to transform relatively low-risk-level areas into low-risk-level areas.

In summary, in the central part of the demonstration zone, human interference is relatively concentrated, but its ecological risk levels are mostly middle, relatively high, and high. This situation indicates that the development of Engebei still faces a contradiction between ecology protection and land use construction. Therefore, there is an urgent need to deal with landscape ecology risks from the perspective of sustainable development.

### Spatial correlation analysis of landscape ecological risks

Lin and Wang [49] evaluate the landscape ecological risks of mountainous cities by dividing regional LER (Landscape Ecological Risks) assessment units, while this article conducts an ecological risk assessment of Engebei by dividing small ecological risk zones. The similarity is that the risk grid is used as the evaluation unit.

Spatial autocorrelation analysis can ascertain whether the variables have spatial correlation and the degree of their correlations. The analysis method can be used to analyze the spatiotemporal change features of ecosystem service value [50] and unpack spatiotemporal evolution regulars of landscape ecological risk [51]. In this paper, this approach is evaluated the landscape ecological risk of the Engebei. Through spatial autocorrelation analysis, the spatiotemporal change characteristic and risk distribution of the ecology in Engebei can be obtained intuitively. And the spatial aggregation pattern of ecological risks in Engebei can be demonstrated by LISA analysis [52]. The local autocorrelation index LISA can reflect the geographical phenomena on local regional units and the correlation between a certain attribute value and adjacent attribute values on local units [43]. The global Moran’s I value is all greater than 0, indicating a positive correlation between the ERI of Engebei. Moreover, the values of global Moran’s I decrease and then increase, indicating that the spatial correlation of ERI in Engebei becomes apparent. From the spatial autocorrelation analysis, we conclude that the distribution area of the high-high aggregation type area is expanding. And the area is consistent with the spatial distribution position of the high-risk-level and relatively-high-risk-level areas. These indicate that the total area of high-risk-level and relatively-high-risk-level areas is gradually expanding. However, the high-risk-level area has decreased, manifesting that the risk level of some high-risk areas has been reduced to other risk levels.

## Conclusions

Since 1980, people have been increasingly investing in the reconstruction of Engebei. We use landscape ecology risk assessment to provide a comprehensive perspective on land use in typical ecologically vulnerable areas under human interference. The conclusions are as follows: (1) The central section of the demonstration area has a high-level landscape ecological risk. And the risk level gradually decreases toward the north and south. The relatively low-risk-level area is spread widely in most of the south and a small part of the north. (2) The selected six landscape pattern indexes can comprehensively demonstrate the relatively distinct diffusion of land types in the demonstration area. (3) The ecological risk values in Engebei have a significant spatial correlation, and the spatial distribution shows a clustering effect, which is consistent with the spatial distribution. The results can provide references for land use planning and construction in similar vulnerable areas. In addition, it is conducive to formulating ecological protection and management policies that are in line with the characteristics of the ecological and economic development of the area.
